# Tunable optical low-pass filtering based on chirped grating in arrays of graphene

**DOI:** 10.1371/journal.pone.0346777

**Published:** 2026-04-29

**Authors:** Yingquan Ao, Xiaoling Chen, Miaomiao Zhao, Hao Ni, Dong Zhong, Fangmei Liu, Xuewen Chen, Junjie Dong, Zhe Liu

**Affiliations:** 1 School of Electronic and Information Engineering, Hubei University of Science and Technology, Xianning, China; 2 Optical Electromechanical and Intelligent Manufacturing Laboratory, Hubei University of Science and Technology, Xianning, China; 3 School of Clinical Medicine, Hubei University of Science and Technology, Xianning, China; Beni-Suef University, EGYPT

## Abstract

We numerically investigate asymmetric light propagation in chirped gratings with embedded graphene arrays (CG-GA). The structure comprises alternating graphene sheets and dielectric layers, featuring a linearly chirped spatial period. Using the forward transmission matrix method (FTMM), we demonstrate that CG-GA support multimode resonance. The transmittance of resonant modes exhibits a significant wavelength-dependent decay, ultimately reaching a cutoff. This phenomenon indicates the structure functions as a tunable optical low-pass filter, whose transmittance, cutoff wavelength, and bandwidth can be modulated by varying the number of grating periods, the chirped period gradient, and the graphene chemical potential. This study may be utilized for spectral scanning and adaptive optics applications.

## 1 Introduction

Photonic crystals are artificial optical structures composed of materials with var-ying refractive indices arranged in accordance with specific rules, offering advantages such as compact size, flexible structural design, high integration, and low loss [1,2]. The most prominent optical characteristic of photonic crystals is the photonic bandgap structure. By meticulously designing the structure of photonic crystals, one can achieve desired optical transmission passbands and stopbands based on the properties of the photonic bandgap, thereby effectively controlling and manipulating the propagation behavior of light. This characteristic provides a theoretical foundation and design guidance for the development of high-performance novel optical devices, such as loss-less optical waveguides [3], optical filters [4,5], optical switches, and optical absorbers [6–8]. Introducing specific dielectric materials at strategic positions within photonic crystals can disrupt their inherent periodic structure, thereby inducing defects in the photonic crystals. These defects can locally confine a substantial number of photons, thereby significantly enhance the photonic density of states, and consequently form new defect modes in the transmission spectrum of photonic crystals or broadening the stopband of photonic crystals. This enables light within a specific frequency range to propagate through the photonic crystal, or inhibits the propagation of light across a broader frequency range. Consequently, leveraging this characteristic of photonic crystals facilitates precise control of light, thereby offering the potential to replace electrons with photons for the transmission, processing, and storage of information. Presently, research on photonic crystal filters predominantly concentrates on tunable multi-channel filters [9,10], two-dimensional photonic crystal channel-drop filters [11,12], and photonic crystal tuning filters [13,14].

Graphene, a novel material that has garnered considerable attention in recent years, is composed of carbon atoms forming a hexagonal honeycomb lattice via hybridized orbitals. This unique lattice structure confers upon it a range of exceptional physical properties, particularly with regard to its electronic band structure, thereby yielding remarkable optoelectronic performance, including extremely high mechanical strength, excellent conductivity, broad spectral absorption range, and very high electron mobility. The thickness of graphene is approximately 0.34 nm. Although the light absorption rate of a single layer of graphene is merely 2.3%, and there are challenges associated with large-scale, high-quality transfer and preparation, these issues can be effectively mitigated through the ingenious design of graphene photonic crystal device structures. Consequently, research on graphene optoelectronic devices possesses considerable theoretical value and promising application prospects [15–23]. Graphene arrays typically refer to graphene layers arranged vertically or at specific angles on a substrate, thereby forming an orderly structure. This arrangement can markedly enhance certain physical properties of graphene, such as thermal and electrical conductivity [24,25]. These crystals also exhibit photonic bandgap structures within the wave vector space. Compared with single-layer graphene, photonic crystals composed of graphene arrays exhibit more pronounced light wave resonance [26], thereby resulting in stronger reflected light within the stopband. Consequently, the bandgap edges be-come steeper, and the extinction effects at these edges are enhanced. Therefore, it is necessary to study the photonic bandgap characteristics of photonic crystals based on graphene arrays.

Chirped gratings are a special type of photonic crystal, the spatial period of which varies with position. This variation can be linear, nonlinear, or other functional forms of change, the grating structure can be regarded as an ordered combination of multiple uniform gratings with different periods [27,28]. By employing deep learning methods, accurate prediction of the transmission spectra of chirped grating structures is achieved [29]. The absorption and localization characteristics of chirped gratings are also analyzed [30,31]. Grating structures are widely used as sensors, filters, circulators, and lasers [32–36]. A chirped gradient photonic crystal resonator suitable for biosensing has been proposed [32]. The reflection spectra, field localization, photonic density of states, and absorption characteristics of photonic crystals under different chirping parameters are analyzed to realize a white-light laser [33]. A graphene-silicon hybrid tunable grating structure is proposed, in which the wavelength and bandwidth are controlled by changing the chemical potential of graphene [34]. In [35], two-dimensional (and even three-dimensional) chirped photonic crystal filter structures are analyzed. Continuous wavelength tuning of a laser is realized by adjusting the chirped fiber Bragg grating [36]. Therefore, it inspires our interest to explore optical propagation characteristics of chirped gratings in graphene arrays.

Building on this foundation, by judiciously selecting the structural medium and parameters, we embedded a monolayer of graphene material into a photonic crystal with an aperiodic arrangement to construct a chirped grating structure model containing graphene arrays. Utilizing the forward transmission matrix method (FTMM), we investigated its optical transmission characteristics and elucidated the variation patterns of the transmission peak position, width, transmittance, and the wavelength corresponding to the cutoff of the transmission peak with respect to factors such as the number of spatial periods of the chirped grating, the spatial period, and the chemical potential of graphene. This approach enabled effective control of the transmission peaks. Compared with traditional dielectric filters, the core advantage of our device lies in its dynamic reconfigurability: By tuning the chemical potential of graphene, continuous tuning of the cutoff wavelength in the mid-infrared to terahertz band (3–35 μm) is achieved, with a tuning rate of －92 nm/mV (Regime I) and －14 nm/mV (Regime II). Functionally, this addresses the limitation of fixed bandwidth in traditional dielectric filters. Although the Q-factor (37–108) of our device is lower than that of high-end multilayer film filters, its tunable range offers unique value for spectral scanning and adaptive optics applications.

## 2 Structure and method

**Fig 1:**
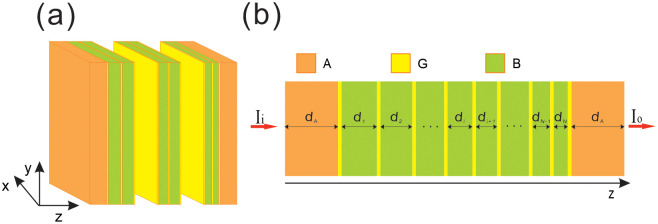
(a) Schematic of chirped grating in graphene arrays. (b) Sideview. The entire structure is represented as $AGB_{\text{1}}GB_{\text{2}}G{...GB_{i}GB_{i+\text{1}}G...GB_{N-\text{1}}GB}_{N}GA$.

The proposed CG-GA is depicted in Fig 1. *G* denotes graphene, dielectric layer *A* is situated on both the incident and exit sides, and dielectric layer *B* is positioned between the graphene arrays of the chirped grating, with each layer of dielectric *B* sequentially numbered from left to right as *B*_1_, *B*_2_, …, *B*_*N*_. Dielectric layers *A* and *B* may be composed of the same material or different materials. Here, the materials used for dielectrics *A* and *B* in this case are both silicon dioxide, with a refractive index of $n_{a}$ = $n_{b}$ = 1.449. The thickness of dielectric layer *A* is denoted as $d_{A}$, and the thickness of the *i*th dielectric layer *B* (i.e., *B*_*i*_) is represented as $d_{i}$. *N* represents the spatial period of the chirped grating, which corresponds to the number of dielectric layers *B*, and the difference between two adjacent spatial periods is a constant, expressed as $d_{i}$ – $d_{i+\text{1}}$ = ${\Delta d}_{var}$. As a transverse wave with sufficiently low intensity impinges upon the system from the left and propagates along the *Z*-axis, the third-order nonlinear effect in graphene can be ignored. The symbol $I_{o}$ represents the transmitted light intensity, while $I_{i}$ denotes the incident light intensity.

Graphene, an ultra-thin two-dimensional nanomaterial, is commonly modeled as a dielectric sheet with a uniform equivalent dielectric constant and an equivalent thickness of $d_{g}$. The equivalent permittivity of graphene is given by [16,26]:


$$\varepsilon_{g}=\text{1}+i\frac{\sigma \eta_{\text{0}}}{k_{\text{0}}d_{g}},$$ (1)

where$\;k_{\text{0}}=\text{2}\pi /\lambda$ is the free-space wave vector, $\eta_{\text{0}}\;$ is the free-space optical impedance, and *σ* is the total surface conductivity of graphene, i is the imaginary unit.

Considering the nonlinear effects of graphene, its surface conductivity [16,26] can be expressed as two components


$$\sigma =\sigma_{\text{1}}+\sigma_{\text{3}}{\left|E_{z}\right|}^{\text{2}},$$ (2)

where $\sigma_{\text{1}}$ represents the linear surface conductivity, $\sigma_{\text{3}}$ represents the nonlinear surface conductivity coefficient, and $E_{z}$ is the electric field component parallel to the plane of incidence.

Graphene’s surface conductivity is governed by the Kubo formula [25,37], with its value dependent on the incident wavelength λ, graphene chemical potential μ, temperature *T*_*g*_, and momentum relaxation time τ. The full mathematical expression is given by:


$$\sigma_{\text{1}}(\omega ,\mu ,\tau ,T_{g})=-\frac{ie^{\text{2}}(\omega +i\tau^{-\text{1}})}{\pi \hbar^{\text{2}}}[\int_{-\infty }^{+\infty }\frac{\left|\varphi \right|}{(\omega +i\tau^{-\text{1}}{)}^{\text{2}}}\frac{\partial f_{d}(\varphi )}{\partial \varphi }d\varphi -\int_{\text{0}}^{+\infty }\frac{\partial f_{d}(-\varphi )-\partial f_{d}(\varphi )}{(\omega +i\tau^{-\text{1}}{)}^{\text{2}}-\text{4}(\varphi /\hbar {)}^{\text{2}}}d\varphi ],$$ (3)

Here, φ denotes the particle energy, *e* represents the elementary charge of an electron, *i* is the imaginary unit, ω stands for the angular frequency, *ћ* is the reduced Planck constant, and *K*_*B*_ is the Boltzmann constant. The function $\frac{f_{d}(\varphi )=\text{1}}{\left(\text{1}+\text{exp}[\frac{\left(\varphi -\mu \right)}{K_{B}T_{g}}]\right)}$ corresponds to the Fermi-Dirac distribution function.

Graphene’s linear surface conductivity $\sigma_{\text{1}}$ consists of two contributions: interband transition conductivity ($\sigma_{inter}$) and intraband transition conductivity ($\sigma_{intra}$). The first term in Equation (3) describes the intraband electron-photon scattering process; integrating this term gives the expression:


$$\sigma_{intra}=i\frac{e^{\text{2}}k_{B}T}{\pi \hbar^{\text{2}}(\omega +i\tau^{-\text{1}})}[\frac{\mu }{k_{B}T}+\text{2}ln(exp(-\frac{\mu }{k_{B}T_{g}})+\text{1})].$$ (4)

The second term in Equation (3) corresponds to interband electron transitions. Under the condition that ℏω and ∣μ∣ are both much larger than *K*_*B*_*T* (i.e., ℏω, ∣*μ*∣ ≫ *K*_*B*_*T*_*g*_), this term can be simplified as


$$\sigma_{\text{inter}}=i\frac{e^{\text{2}}}{\text{4}\pi \hbar^{\text{2}}}ln\left[\frac{\text{2}|\mu |-\hbar \left(\omega +i\tau^{-\text{1}}\right)}{\text{2}|\mu |+\hbar \left(\omega +i\tau^{-\text{1}}\right)}\right].$$ (5)

From the above expression, the conductivity arising from both interband and intraband transitions is dependent on two key parameters: graphene’s chemical potential and the photon frequency. Within the terahertz band, photon energy is relatively low; specifically, when ℏ*ω* < 2*μ* holds, intraband transitions become the dominant contribution to conductivity.

The graphene-array-based chirped grating is oriented along the Z-axis, with light normally incident on the structure’s left face and transmitted out from the right. Excluding optical nonlinear effects, the linear transmission and reflection spectra of this periodic configuration are computed via the Forward Transfer Matrix Method (FTMM) [8,25]. Graphene’s chemical potential corresponds to its Fermi level, which can be tuned via chemical doping or external gate voltage: a positive bias raises the chemical potential, while a negative bias lowers it. This allows flexible adjustment of graphene’s surface conductivity through applied bias voltages. Additionally, some other parameters are set Table 1.

**Table 1:** Basic parameters.

Parameter	Symbol	Value
Graphene chemical potential	*μ*	0.2-0.5 eV
Environment temperature	*T*_*g*_	300 K
Equivalent thickness of graphene layer	*d*_*g*_	0.33 nm
Relaxation time	*τ*	0.5ps (0.2–0.5 ps)
Refractive Indices	*n*	1.449
Incident angle	*θ*	0-45°
Number of Periods	*N*	20(20-40)
The difference between two adjacent spatial periods	${\Delta d}_{var}$	10-40 nm

The reflectance and transmittance of light waves can also be derived by the FTDT (finite-different time-domain), which is a numerical solution and the calculating precision depends on the step of sampling points. It means that, to obtain high precision results requires more hardware resources or costs more time by the FTDT. However, the reflectance and transmittance are analytical solutions by the transfer matrix method as light waves propagates in multilayers, and the entire structure can be viewed as a cascaded system constituted by a battery of dielectrics (graphene can also equates to a dielectric with an equivalent thickness), then total transfer matrix of the whole structure is expressed as a product of the transfer matrices of homogeneous layers.

## 3 Results and discussion

### 3.1 Optical spectra vs. grating periods, spatial periods, and chemical potential in CG-GA

**Fig 2:**
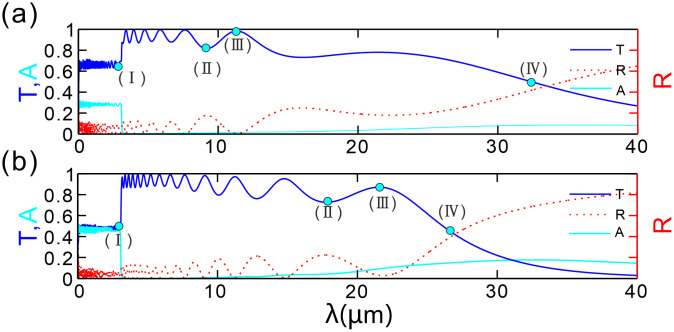
(a-b) Transmission (*T*), absorption (*A*) and reflection (*R*) spectra corresponding to the spatial periods of the chirped grating with *N* = 20 and 40, respectively. The graphene chemical potential is *μ* = 0.2 eV, the thickness difference between two adjacent layers of type-*B* dielectric is ${\Delta d}_{var}$ = 10 nm.

When a plane wave is incident perpendicularly along the horizontal direction onto the chirped grating structure, the reflectance and transmittance vary with different chirped grating periods *N*. In this study, the FTMM is employed. Fig 2 illustrates the transmittance and reflectance curves corresponding to different chirped grating periods *N*. Assuming the input wave is a transverse electric wave incident perpendicularly and neglecting nonlinear effects, the chemical potential of graphene is set at *μ* = 0.2 eV (where eV denotes electron-volt) and *d*_*A*_ = 2 μm.

Fig 2(a) presents the optical transmission spectrum of the structure, where the transmittance (*T*, blue solid line), absorbance (*A*, cyan solid line), and reflectance (*R*, red dashed line) are plotted as functions of wavelength in the range of 0–40 μm. Within the wavelength interval of 0–20 μm, the transmission spectrum exhibits a series of resonance peaks, and the spacing between adjacent peaks gradually widens with increasing wavelength. A significant upward jump in transmittance occurs at the jump wavelength *λ*_*jump*_ = 3.1 μm, where the value abruptly increases from 0.69 to approximately 0.87. When the wavelength exceeds 3.1 μm, the transmittance approaches 1 and fluctuates slightly around this value. When short-wavelength light impinges on the structure, interband transitions of electrons dominate, resulting in significant attenuation of the light wave intensity; in contrast, long-wavelength incident light primarily triggers intraband transitions. With a further increase in wavelength, the transmittance decreases rapidly, and the transmission curve demonstrates a distinct cutoff behavior.

Here, the cutoff wavelength is defined as the longer wavelength corresponding to the 50% reduction in the peak transmittance of the last clearly resolvable resonance peak within the passband, which corresponds to a 3 dB stopband rejection ratio. The effective bandwidth of the filter is defined as the wavelength interval between the passband jump point and the cutoff point. As denoted by the cyan dots in the Fig 2: (Ⅰ) the jump point with the minimum transmittance in the passband; (Ⅱ) the point with the local minimum transmittance within the effective bandwidth; (Ⅲ) the point with the maximum transmittance of the last resonance peak in the passband; (Ⅳ) the cutoff wavelength point. The cutoff wavelength is determined to be approximately 33 μm. The reflectance exhibits a complementary relationship with the transmittance: transmission peaks correspond to reflection dips. As the wavelength increases and transmission attenuates, reflection strengthens. When *λ* ＜ *λ*_*jump*_, the absorption peak reaches 0.3 due to the interband transition of graphene. When *λ* ＞ *λ*_*jump*_, the absorption remains below 0.1. Verification of energy conservation (*R* + *T* + *A* ≈ 1) at the marked points (Ⅰ-Ⅳ) shows errors all less than 1%.

As evidenced by Fig 2(b), when the number of periods (*N*) increases from 20 to 40, the density of resonance peaks increases, and the peak transmittance decays with wavelength. At point (Ⅰ) (*λ*_*jump*_ = 3 μm), the transmittance jumps from 0.69 to 0.87. Notably, the transition band width of *N* = 40 (9.15 μm) is only 38.3% of that of *N* = 20 (23.875 μm). This result indicates that increasing the number of periods not only causes a blue shift of the cutoff wavelength but also significantly enhances the attenuation rate of transmittance from the “stable passband” to the “stopband.” For the *N* = 20 structure, a stopband wavelength of 39.5000 μm (6.8750 μm after the 3 dB cutoff wavelength of 32.6250 μm) is selected, with a simulated transmittance of 0.278746, corresponding to a stopband rejection ratio of approximately 5.5 dB. For the *N* = 40 structure, a stopband wavelength of 36.0625 μm (9.2500 μm after the 3 dB cutoff wavelength of 26.8125 μm) is chosen, and its simulated transmittance is 0.060819, leading to a stopband rejection ratio of about 11.6 dB.

Within the graphene array, numerous resonant cavities exist. When the wavelength of the incident light wave precisely satisfies the resonant condition, a series of resonance peaks are induced on the transmission spectrum curve. As the wavelength of the incident light increases and no longer satisfies the resonant condition, coupled with the effect of the surface current of graphene, the transmittance drops sharply, leading to a cutoff, thereby suppressing long wavelengths. Consequently, this chirped grating can be utilized in optical low-pass filters. By adjusting the structural parameters, one can flexibly control the bandwidth and cutoff characteristics of the filter. This level of tunability is not available in traditional optical filters, offering significant versatility for practical applications.

**Fig 3:**
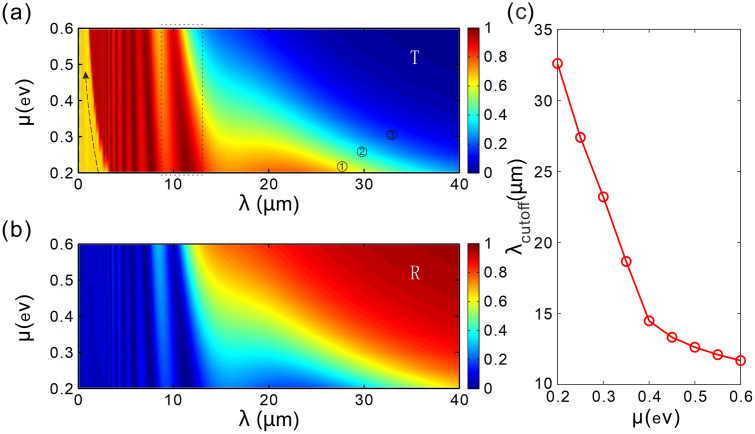
(a) The relationship between transmittance and the incident wavelength, as well as the graphene chemical potential. (b) The relationship between reflectance and the incident wavelength, as well as the graphene chemical potential. (c) Relationship between the cutoff wavelength and the chemical potential of graphene. The number of spatial periods of the chirped grating is *N* = 20, the thickness difference between two adjacent layers of type-*B* dielectric is ${\Delta d}_{var}$ = 10 nm.

Given that the chemical potential is a key parameter influencing the surface conductivity of graphene, the equivalent permittivity of graphene is intrinsically dependent on the chemical potential. This dependence enables the modulation of the transmission performance by tuning the chemical potential of graphene in a flexible manner. For various values of chemical potential, Fig 3 shows the variation of transmission and reflection spectra with the chemical potential. From Fig 3(a), it can be observed that the transmittance curve is influenced by the graphene chemical potential. At shorter wavelengths, the transmittance undergoes a sudden change, and as the graphene chemical potential increases, the wavelength at which this sudden change occurs shifts towards shorter wavelengths (as indicated by the dashed arrow). As the incident wavelength increases, it can be observed that the transmission peaks become broader, while the transmission decreases with increasing chemical potential. On the right side of the rectangular dashed box, when the wavelength continues to increase, the higher the graphene chemical potential, the more rapidly the transmission is cut off. For example, as shown in the Fig 3, curve ① corresponds to a smaller chemical potential compared to curves ② and ③, resulting in a shorter cutoff wavelength, i.e., a narrower bandwidth. Consequently, we can flexibly adjust the cutoff wavelength and, by extension, the bandwidth of the filter by modulating the chemical potential of graphene. This capability allows the filter to be tailored to various communication band requirements. In the mid-infrared range (3–20 μm), if the resonance peaks and cutoff wavelengths fall within this spectrum, precise control over specific wavelength ranges within this band can be achieved by adjusting the chemical potential of graphene. For instance, increasing the chemical potential can alter the position of the resonance peaks and the transmittance’s cutoff wavelength, thereby enabling filtering of light waves in the mid-infrared spectrum.

Fig 3(b) illustrates the resonance-stopband transition characteristics of the chirped grating, where chemical potential tuning effectively modulates the resonance positions. In the long-wavelength region (*λ* > 32 μm), the curves converge to *R* ≈ 0.6–0.8, forming a high-reflectivity plateau that indicates entry into the stopband. In the passband region (*λ* < 12 μm), the curves exhibit dense multi-mode resonance with multiple reflection minima. Within the transition band (12–32 μm), the edge positions of the reflectivity are regulated by the chemical potential *μ*.

Fig 3(c) shows the segmented tuning characteristics of the chirped graphene grating. The cutoff wavelength *λ*_cutoff_ exhibits two linear regimes: Regime I (*μ* < 0.35 eV, slope = －92 *μ*m/eV, coefficient of determination *R*^2^ = 0.998) and Regime II (*μ* > 0.35 eV, slope = －14 *μ*m/eV, coefficient of determination *R*^2^ = 0.958). Our device achieves dynamic tuning in the mid-infrared to terahertz band (3–35 μm), with a tuning rate of －92 nm/mV (Regime I) and －14 nm/mV (Regime II), functionally addressing the limitation of fixed bandwidth in traditional dielectric filters.

**Fig 4:**
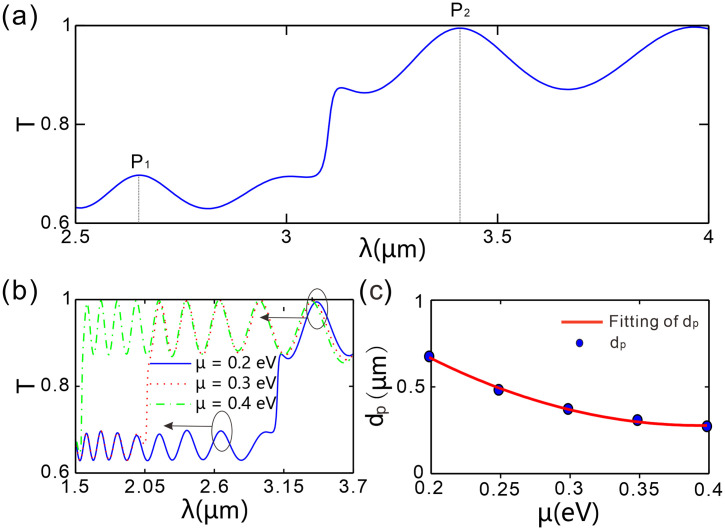
(a) Local transmission spectrum when *μ* = 0.2 eV. (b) The transmission spectrum corresponding to peak $P_{\text{1}}$ and peak $P_{\text{2}.}$ (c) Peak-to-peak spacing ${𝐝}_{p}$ as a function of graphene chemical potential. The number of spatial periods of the chirped grating is *N* = 20, the thickness difference between two adjacent layers of type-*B* dielectric is ${\Delta d}_{var}$ = 10 nm.

Fixing the period number, a portion of the transmission spectrum (*λ* ∈ [2.5 μm, 4 μm]) from Fig 2(a) was analyzed to investigate the variations of two resonance peaks (peak $P_{\text{1}}$ before the jump and peak $P_{\text{2}}$ after the jump) with the graphene chemical potential in Fig 4(a). The spacing between the resonant wavelengths of *P*₁ and *P*₂ is denoted as $d_{p}$, as indicated by the two dashed lines. The P1 and P2 peaks represent the Bragg diffraction resonances of different orders (or corresponding to different local periods) supported by this chirped grating structure. As shown in Fig 4(b), peak *P*₁ (the resonance peak before the jump) locates in the short-wavelength region (*λ* **＜** *λ*_***jump***_), where interband transitions of graphene dominate. At this point, graphene exhibits metallic-like characteristics, leading to increased losses and thus reduced transmittance, so *P*₁ appears as a “weak transmission peak.” Interband transitions occur only when the incident photon energy satisfies *ℏω* > 2*μ*, a process that induces significant optical loss. Consequently, an increase in the chemical potential raises the lower limit of the photon frequency required for interband transitions, thereby shifting the transmittance (*T*) curve towards shorter wavelengths. In contrast, peak *P*₂ (the resonance peak after the jump) lies in the long-wavelength region (*λ* **＞** *λ*_***jump***_), where intraband transitions dominate. In this region, the optical absorption of graphene is significantly reduced, so *P*₂ appears as a “strong transmission peak.” Its wavelength is mainly determined by the geometric parameters of the grating (chirped period distribution), and the regulatory effect of the chemical potential on *P*₂ is weaker than that on *P*₁.

On the other hand, we analyze the evolution of peak stability (including width, sharpness, and intensity) and the quality factor *Q* with respect to the graphene chemical potential. Peak *P*_1_ (T_peak ≈ 0.68-0.70) and peak *P*_2_ (T_peak ≈ 0.97-0.99) exhibit regular variations in their stability parameters as *μ* increases: the peak width (full width at half maximum, FWHM) shows an overall monotonic reduction (P1 decreases from 0.0700 μm to 0.0300 μm, and *P*_2_ from 0.1100 μm to 0.0250 μm), while the peak intensity (characterized by T_peak) and peak-to-valley height difference) decreases slightly but remains relatively stable. The evolution of the quality factor *Q* exhibits distinct characteristics: the *Q* value of *P*_1_ increases monotonically with *μ* (from 37.86 to 49.83, corresponding to a 31.6% increase), whereas that of *P*_2_ follows a non-monotonic trend of first increasing sharply and then decreasing slightly (from 31.00 to 108.50 and then to 63.80), peaking at *μ* = 0.3 eV. It is noteworthy that *μ* can tune the peak width, sharpness, intensity, and quality factor *Q*, and exerts a more pronounced regulatory effect on Peak *P*_2_. Unlike graphene-based tunable metamaterial devices [38,39], our device focuses on broad-spectrum continuous cutoff wavelength tuning (3-12 μm), and its moderate *Q*-factor (37-108) is sufficient for practical applications such as spectral scanning and adaptive optics.

Fig 4(c) illustrates the dependence of the peak-to-peak spacing $d_{p}$ on the graphene chemical potential. Data show that as *μ* increases continuously within the range of 0.2 ~ 0.4 eV, $d_{p}\;$ exhibits a stable monotonic decreasing trend: $d_{p}$ reaches its maximum value (0.760 μm) at *μ* = 0.2 eV and its minimum value (0.195 μm) at *μ* = 0.4 eV. Furthermore, the decreasing amplitude of $d_{p}$ gradually slows down with each 0.05 eV increase in *μ.* For instance, $d_{p}$ decreases by 0.270 μm when *μ* increases from 0.2 to 0.25 eV, while it only decreases by 0.050 μm when *μ* rises from 0.35 to 0.4 eV - presenting a “nonlinear decreasing” characteristic. This trend stems from the differential regulation of the two resonance peaks (*P*_1_ and *P*_2_) by *μ*: *P*_1_ (located in the interband transition region) is more strongly affected by *μ*, and its blue-shift amplitude is larger than that of *P*_2_ (situated in the intraband transition region), leading to a continuous reduction in the peak-to-peak spacing as *μ* increases. For the *P*_2_ peak, when *μ* = 0.3 eV, it achieves a quality factor (*Q*) of 108.5 and a full width at half maximum (FWHM) of 0.025 μm (25 nm), which meets the requirements of optical communication technology [40,41]. Additionally, the peak spacing *dₚ* can be continuously tuned from 0.760 μm to 0.195 μm, enabling dynamic reconfiguration of the filter bandwidth [42,43]. These characteristics provide value for adaptive optics applications.

**Fig 5:**
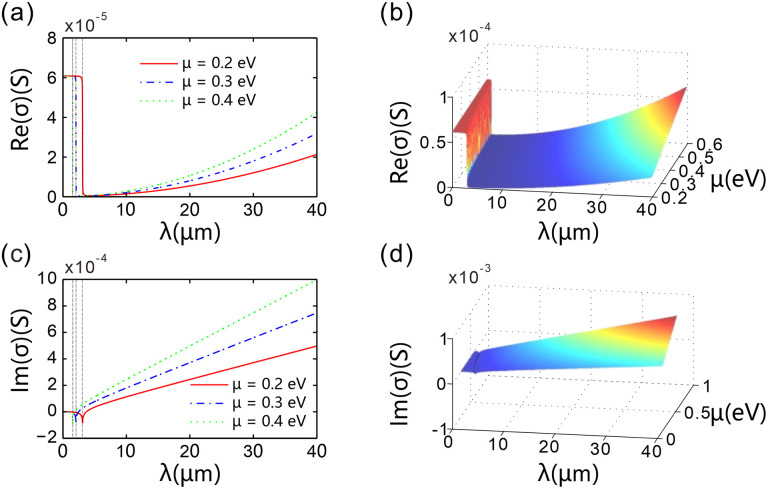
(a, c) Plots of the real part Re(σ) and imaginary part Im(σ) of the surface conductivity σ as a function of the incident wavelength for various fixed chemical potentials of graphene. (b, d) Variations of Re(σ) and Im(σ) with respect to the chemical potential and the incident wavelength. The number of spatial periods of the chirped grating is *N* = 20, the graphene chemical potential is *μ* = 0.2 eV.

To investigate the formation mechanism of these jumps in the transmission spectrum, this paper examines the correlation between the incident wavelength and the linear surface conductivity of graphene. For three distinct chemical potentials *μ* = 0.2 eV, 0.3 eV, and 0.4 eV. Fig 5a presents the real part Re(*σ*) of graphene’s surface conductivity: For each curve, at shorter incident wavelengths, Re(*σ*) approximates 6.1 × 10^−5^ S and remains constant as the wavelength increases. However, as the wavelength continues to rise, Re(*σ*)drops sharply; with further increases in wavelength, this real part stays near zero over a certain interval. When the wavelength increases beyond 12 μm (for instance), Re(*σ*) begins to increase again. Directly derived from the photon energy-wavelength formula, the final relation is obtained as $\lambda =\frac{\hbar \text{c }}{\text{2}\mu }$. Specifically, when *μ* = 0.2 eV, λ = 3.1 μm; when *μ* = 0.3 eV, λ ≈ 2.07 μm; and when *μ* = 0.4 eV, λ = 1.55 μm. This wavelength serves as the demarcation point between the interband transition (ℏ*ω* > 2*μ*) and intraband transition (ℏ*ω* ＜ 2*μ*) of graphene. This paper treats graphene as a dielectric with an equivalent thickness; thus, Re(*σ*) corresponds to the imaginary part of $\varepsilon_{g}$ (the optical loss term). A larger Re(*σ*) indicates stronger optical absorption loss in the device, resulting in lower transmittance and higher reflectance. However, for the spectral cutoff phenomenon observed in the long-wavelength region (33 μm) in Fig 2, although Re(σ) gradually increases with increasing wavelength in this band (reaching approximately 2.1 × 10 ⁻ ⁵ S at 40 μm), indicating that the intrinsic absorption loss of graphene is not the primary factor, the transmittance drops sharply to zero. This phenomenon is attributed to the fact that when the light wavelength exceeds the phase matching limit of the grating, the system enters an interval dominated by radiation leakage and the absorption loss and dielectric loss of graphene. The light energy cannot be effectively transmitted but is rapidly dissipated through the above-mentioned mechanism, resulting in a sharp cut-off of the transmission spectrum.

Fig 5(b) gives Re(*σ*) variation in the parameter space composed of chemical potential and incident wavelength. On the left side of the parameter space, Re(*σ*) varies with the change in wavelength and the chemical potential. Near the parameters of *μ* = 0.2 eV and *λ* = 2.75 μm, Re(*σ*) undergoes a mutation. This is the result of the transition from intraband to interband transitions of electrons in graphene. As *μ* increases, the incident wavelengths at which the real part of the surface conductivity of graphene changes sharply will blue-shift (shifting towards shorter wavelengths). On the right side of the parameter space, specifically the blue sheet-like area, the value of Re(*σ*) can be increased by increasing the chemical potential of graphene or by increasing the incident wavelength.

Fig 5(c) illustrates the imaginary part of the surface conductivity Im(*σ*) changing with the wavelength of the incident light. For different values of *μ* = 0.2 eV, 0.3 eV, and 0.4 eV, there are three corresponding curves, each with a minimum that blue-shifts as *μ* increases. However, due to the ultra-thin nature of graphene, this distinct feature has a minimal impact on the transmittance. Im(*σ*) corresponds to the real part of the equivalent dielectric constant, which primarily influences the effective refractive index of graphene and thereby modifies the reflection phase of light waves at the interface. Due to graphene’s ultra-thin nature, the dips in Im(σ) exert a limited impact on the absolute value of transmittance *T*.

Based on the effective resonance peaks on the right of the *ℏω* = 2*μ* transition threshold (intraband region, Re(*σ*) ≈ 0), we quantitatively analyzed Im(*σ*)’s regulatory effect on optical resonance performance (Table 2). At *μ* = 0.3 eV, Im(*σ*) = －8.847 × 10 ⁻ ⁵ S, the resonance peak exhibits the minimum full width at half maximum (FWHM) of 0.02 μm, with the quality factor (*Q*) reaching a maximum of 108.50. When the absolute value of Im(*σ*) deviates from this optimal value (－8.539 × 10 ⁻ ⁵ S at *μ* = 0.2 eV and －9.878 × 10 ⁻ ⁵ S at *μ* = 0.4 eV), the FWHM increases to 0.025 ~ 0.11 μm and the *Q* factor decreases to 31.00 ~ 63.80, demonstrating that Im(σ) is not the sole controlling parameter.

**Table 2:** The corresponding relationship between Im(*σ*) and resonance performance.

*μ(*eV)	λ_jump_(μm)	Im(*σ*) at the threshold	P2 peak position(μm)	FWHM（μm）	Quality factor Q
0.2	3.10	－8.539 × 10 ⁻ ⁵	3.410	0.1100	31.00
0.3	2.07	－8.847 × 10 ⁻ ⁵	2.170	0.0200	108.50
0.4	1.55	－9.878 × 10 ⁻ ⁵	1.595	0.0250	63.80

Fig 5(d) gives Im(*σ*) changing with the chemical potential of graphene and the incident wavelength in the parameter space. On the left side of the parameter space, there is a groove in Im(*σ*). This indicates that the incident wavelength at the minimum of Im(*σ*) blue-shifts with the increase in the chemical potential of graphene. Abrupt changes in the real part of the refractive index of graphene’s equivalent dielectric exert only a slight impact on the transmittance.

**Fig 6:**
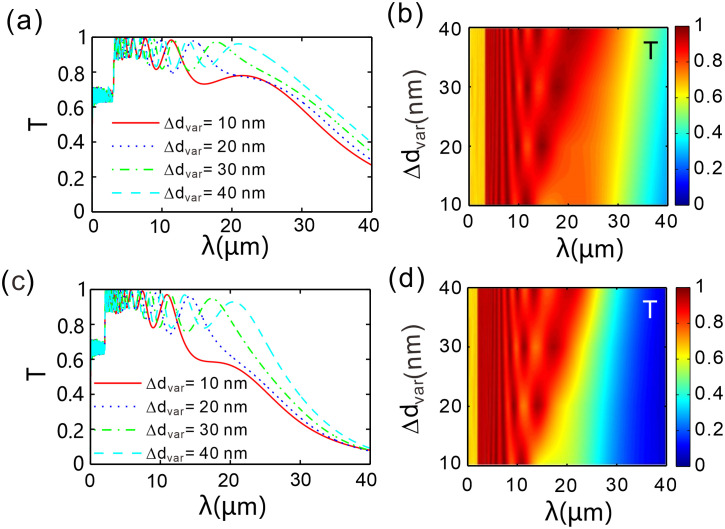
(a, c) Transmission spectra for different spatial periods of the chirped grating at chemical potentials *μ* = 0.2 eV and 0.3 eV; (b, d) Two-dimensional variation of transmittance with incident wavelength and thickness difference ${\Delta d}_{var}$ of adjacent two layers of B-type dielectric at chemical potentials *μ* = 0.2 eV and 0.3 eV. The number of spatial periods of the chirped grating is *N* = 20.

Maintaining *N* = 20, the spatial period of the chirped grating was altered. That is, the total number of dielectric *B* elements is kept constant, while the thickness of each dielectric *B* element is changed. Fig 6 presents the transmittance of this chirped grating. Here, $d_{\text{1}}$ = 500 nm, $d_{\text{2}}$ = 480 nm, …, $d_{N}$ = 100 nm are considered, with the thickness difference between adjacent layers of type-*B* dielectric being ${\Delta d}_{var}$ = 20 nm, while other parameters remain unchanged. Figs 6(a) and 6(c): As ${\Delta d}_{var}$ increases from 10 nm to 40 nm, the resonance mode density is significantly enhanced, with the number of transmission dips rising from approximately 6–12, while the 3 dB bandwidth of each dip broadens monotonically with wavelength. A comparison between Fig 6(a) and 6(c) reveals that the resonance contrast at *μ* = 0.3 eV is markedly higher than that at *μ* = 0.2 eV, indicating that an increased chemical potential strengthens the grating modulation depth. Figs 6(b) and 6(d): In the low-loss region at *μ* = 0.2 eV, the increase in ${\Delta d}_{var}$ leads to a broadening of the passband (*T* > 0.7), manifested as the horizontal expansion of the red region, where the chirp effect dominates. Conversely, at *μ* = 0.3 eV, the imaginary part of the graphene conductivity increases substantially, and losses suppress the passband transmittance; despite a higher resonance mode density, the effective passband width contracts.

### 3.2 Transmittance spectra vs. momentum relaxation times and incident angle in CG-GA

**Fig 7:**
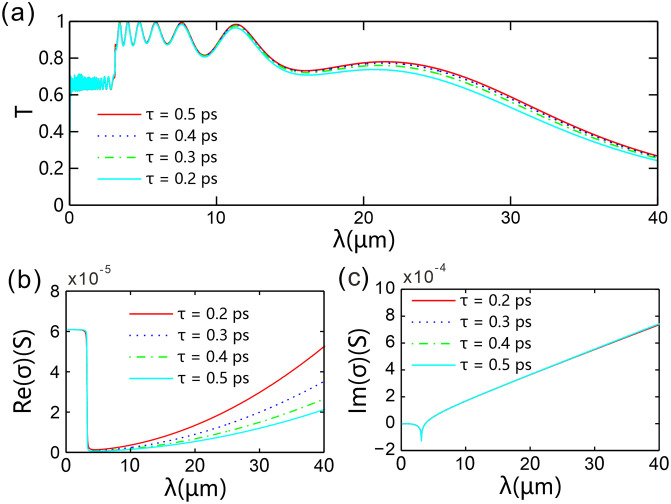
(a) Transmission spectra versus different relaxation time *τ.* (b, c) Real part and imaginary part of graphene surface conductivity varying with *τ*. The number of spatial periods of the chirped grating is *N* = 20, the thickness difference between two adjacent layers of type-*B* dielectric is ${\Delta d}_{var}$ = 10 nm, the graphene chemical potential is *μ* = 0.2 eV.

The surface conductivity of graphene is a function of the parameter *τ*, which is defined as the phenomenological relaxation time of electrons in graphene. Fig 7(a) presents the transmission spectra under different momentum relaxation times *τ* (ranging from 0.2 ps to 0.5 ps). It can be observed that the core structural characteristics of the transmission spectrum remain essentially unchanged with variations in *τ*: the resonance peak on the far right of the target operating range only exhibits a slight drift within 11.300–11.330 μm (relative deviation < 0.27%), the full width at half maximum (FWHM) of the resonance mode is stably maintained at 0.1800 μm, and the cutoff wavelength fluctuates moderately between 31.395 μm and 32.590 μm (relative deviation < 3.7%). Meanwhile, the peak-valley height difference of the resonance peak remains approximately 0.0049 (only slightly decreasing to 0.0048 when *τ* = 0.2 ps). However, overall, the resonance peak in the transmission spectrum is significantly enhanced as *τ* increases: the peak transmittance rises from 0.9649 at *τ* = 0.2 ps to 0.9834 at *τ* = 0.5 ps, and the corresponding quality factor *Q* gently increases from 62.78 to 62.94. A monotonic decrease of 0.25% confirmed that the energy transmission quality is robust to τ variation within this interval. This trend originates from the reduction in optical loss of graphene with the increase in momentum relaxation time *τ*. Notably, *τ* only modulates the surface conductivity of graphene (thereby affecting optical loss) and does not alter the geometric parameters of the chirped grating. Consequently, the passband position, resonance mode width, and cutoff wavelength in the transmission spectrum are not significantly affected by *τ*.

Fig 7(b) presents the real part of the surface conductivity, Re(*σ*), for different values of *τ*: for the four values of *τ* = 0.2–0.5 ps, the Re(*σ*) values in each corresponding curve vary with wavelength. Re(*σ*) determines the optical loss of graphene; therefore, for a fixed *τ* in the core operating region, Re(*σ*) increases with increasing wavelength, and as a result, as shown in Fig 7(a), the transmittance decreases as the wavelength increases. Furthermore, at a fixed incident wavelength, the smaller *τ* is, the larger Re(*σ*) is, which means it will cause more significant optical loss. Fig 7(c) shows that the imaginary part of the surface conductivity, Im(*σ*), is not affected by *τ*: for different *τ* values in the core operating region, Im(*σ*) increases with increasing wavelength, and the four surface conductivity curves corresponding to *τ* = 0.2–0.5 ps almost completely overlap. Since Im(*σ*) modulates the real part of the equivalent permittivity of graphene, modulating the phenomenological relaxation time cannot change the bandgap characteristics in the transmission spectrum.

**Fig 8:**
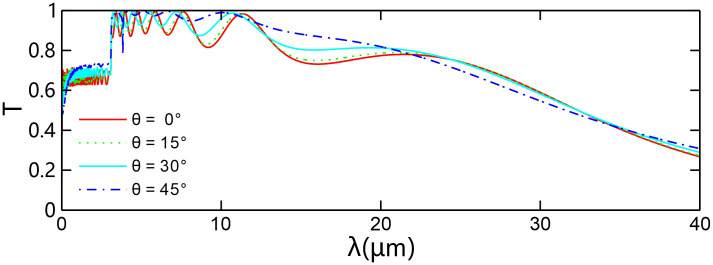
Transmission spectra varying with the incident wavelength for some given incident angles. The number of spatial periods of the chirped grating is *N* = 20, the thickness difference between two adjacent layers of type-*B* dielectric is ${\Delta d}_{var}$ = 10 nm, the graphene chemical potential is *μ* = 0.2 eV.

For four given angles of incidence, Fig 8 illustrates the corresponding transmission spectra. The results indicate that when *λ* < 3 μm, the height of the transmission peaks gradually increases with the increase in the angle of incidence; when *λ* ≥ 3 μm, the transmission spectrum curve as a whole blue-shifts with the increase in the angle of incidence. Consequently, the position of the bandwidth in the transmission spectrum can also be adjusted by changing the angle of incidence. For a fixed wavelength, as the angle of incidence increases, the horizontal component of the corresponding wave vector decreases, thereby shifting the position of the bandwidth towards shorter wavelengths.

### 3.3 Chirp irregularities and their spectral impact

**Fig 9:**
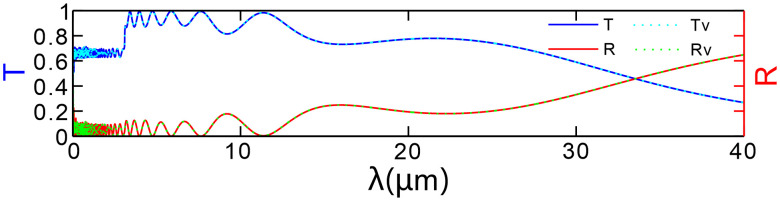
Comparison of transmission and reflection spectra for ${𝐝}_{i}$ and ${𝐝}_{i,theoretical}$.

The periodic parameters of the structure, such as period length and dielectric layer thickness, may not achieve complete uniformity and precision. To simulate the errors that may occur during the manufacturing process, we have adopted a fine-tuning method that introduces periodic irregularities by adjusting the actual thickness of each layer of the medium. We have developed a fine-tuning equation: $d_{i}=d_{i,theoretical}\times (\text{1}+\delta_{i})$, where $d_{i}$ denotes the actual thickness of the *i*-th layer of the medium, $d_{i,theoretical}$ represents the corresponding theoretical design value, and $\delta_{i}$ is a factor representing random or systematic deviation, used to simulate the irregularities in the manufacturing process. For a period, *N* = 20 with $d_{\text{1}}$ = 300 nm, $d_{\text{2}}$ = 290 nm, …, $d_{N}$ = 100 nm as the theoretical data, assuming the deviation is between −5% and +5%. Fig 9 presents a comparison of the transmittance and reflectance results. The solid lines (labeled *T* and *R*) represent the predicted results of the theoretical model, whereas the dashed lines (labeled *T*v and *R*v) represent the simulation results after accounting for the irregularities. It can be observed that the data on transmittance and reflectance align with the theoretical expectations. To evaluate the manufacturing robustness of the device, a normalized sensitivity index S is defined based on the simulation data as: S$=\frac{\left|X_{\text{0}}-X_{v}\right|}{X_{\text{0}}}$, where $X_{\text{0}}$ is the theoretical value of the target optical parameter (cutoff wavelength *λ*_cutoff_, peak transmittance *T*_max_, or resonance peak position *λ*_p_, and $X_{v}\;$is the average simulated value after thickness perturbation. The results demonstrate that the sensitivity indices *S* for *λ*_cutoff_, *T*_max_, and *λ*_p_ are all less than 1%. Specifically, the *S* values for *T*_max_ and *λ*_cutoff_ are below 0.1%, indicating that the device exhibits extremely low sensitivity to thickness errors and possesses industrial-grade manufacturing robustness.

Plasma Enhanced Chemical Vapor Deposition (PECVD) is a mature and widely used technique in micro- and nanofabrication, allowing precise control over the thickness and uniformity of dielectric layers [44]. These dielectric layers are designed according to the arrangement rules of the chirped grating, and the in-situ ellipsometry monitoring ensures a thickness accuracy of ±1 nm, so as to achieve the desired optical properties. Pre-fabricated monolayer graphene (a mature product) can be transferred onto the surface of the prepared metal substrate [45,46]. Additionally, graphene could also be directly deposited on the surface of dielectric layers via CVD [47]. Through a stepwise independent process, the proposed chirped structure exhibits high feasibility for implementation in a laboratory setting.

## 4 Conclusions

In this work, we numerically demonstrate that the chirped graphene-dielectric grating structure exhibits unique multimode resonance characteristics, enabling tunable optical low-pass filtering functionality. By tuning key parameters including the number of grating periods, graphene chemical potential, and spatial period, flexible modulation of the resonance modes, cutoff wavelength, and bandwidth can be achieved. Specifically, continuous tuning of the cutoff wavelength across the mid-infrared to terahertz band (3–35 μm) is realized by adjusting the graphene chemical potential, with tuning rates of −92 nm/mV (Regime I) and −14 nm/mV (Regime II), which effectively addresses the limitation of fixed bandwidth in traditional dielectric filters. Its broad tuning range provides unique value for spectral scanning and adaptive optics applications.
